# Supplementary material for: The socioeconomic impact of orthopaedic trauma: A systematic review and meta-analysis
Source: PLoS One. 2020 Jan 15;15(1):e0227907. doi: 10.1371/journal.pone.0227907 (PMC6961943; doi:10.1371/journal.pone.0227907)
Supplement: S1 File — (DOCX) [file pone.0227907.s001.docx]

**Supporting Information**

**S1 File. Detailed Search Strategies.**

**MEDLINE (Ovid)**

(((socioeconomic OR financial OR economic).ab,ti ADJ1 (impact OR consequence* OR burden OR stress).ab,ti) OR employment.ab,ti OR unemployment.ab,ti OR unemployed.ab,ti OR loss of work.ab,ti OR missed work.ab,ti OR return to work.ab,ti OR sickness absence.ab,ti OR income.ab,ti OR cost of illness/ OR return to work/ OR absenteeism/ OR income/) **AND**

(((hip OR leg OR knee OR arm OR upper extremity OR lower extremity OR limb OR bone OR orthopaedic OR orthopedic) ADJ2 (trauma OR injur* OR fracture*)).ab,ti. OR dislocation.ab,ti. OR orthopaedic surgery.ab,ti. OR orthopedic surgery.ab,ti. OR orthopedics/ OR orthopedic procedures/ OR fractures, bone/ OR hip injuries/ OR leg injuries/ OR knee injuries/ OR arm injuries/)

**Embase**

(((socioeconomic OR financial OR economic) NEXT/1 (impact OR consequence* OR burden OR stress)):ab,ti OR employment:ab,ti OR unemployment:de,ab,ti OR unemployed:ab,ti OR “loss of work”:ab,ti OR “missed work”:ab,ti OR “return to work”:de,ab,ti OR “sickness absence”:ab,ti OR income:ab,ti OR “financial deficit”/de OR “employment status”/de OR ‘income’/de) **AND**

(((hip OR leg OR knee OR arm OR “upper extremity” OR “lower extremity” OR limb OR bone OR orthopaedic OR orthopedic) NEAR/2 (trauma OR injur* OR fracture*)):ab,ti OR dislocation:ab,ti OR “orthopaedic surgery”:ab,ti OR “orthopedic surgery”:ab,ti OR orthopedics/de OR “joint fracture”/exp OR “limb fracture”/exp OR “pelvic fracture”/exp OR “hip injury”/exp OR “hip fracture”/exp OR “leg fracture”/exp OR “knee injury”/exp OR “arm fracture”/exp)

**Scopus** - TITLE-ABS-KEY

((socioeconomic OR financial OR economic) Pre/1 (impact OR consequence* OR burden OR stress) OR employment OR unemployment OR unemployed OR “loss of work” OR “missed work” OR “return to work” OR “sickness absence” OR income) **AND**

((hip OR knee OR leg OR arm OR “upper extremity” OR “lower extremity” OR limb OR bone OR orthopaedic OR orthopedic) W/2 (trauma OR injur* OR fracture*) OR dislocation OR “orthopaedic surgery” OR “orthopedic surgery”)
