# Supplementary material for: The socioeconomic impact of orthopaedic trauma: A systematic review and meta-analysis
Source: PLoS One. 2020 Jan 15;15(1):e0227907. doi: 10.1371/journal.pone.0227907 (PMC6961943; doi:10.1371/journal.pone.0227907)
Supplement: Attachment — Submitted filename: Response to Reviewers.docx [file pone.0227907.s004.docx]

**Response to Reviewers**

**Journal Requirements:**

When submitting your revision, we need you to address these additional requirements.

1. Please ensure that your manuscript meets PLOS ONE's style requirements, including those for file naming. The PLOS ONE style templates can be found at

**Response:** We have adjusted the file naming to comply with PLOS ONE’s requirements.

2. Please ensure you have performed the latest search within the past 12 months, the current search is out of date.

**Response:** We have updated the search on December 3, 2019. The revised search has added 15 new articles to the systematic review and meta-analysis but did not qualitatively change the findings of the study.

**Revision:** An experienced academic research librarian conducted searches in MEDLINE (Ovid), Embase (Elsevier), and Scopus on December 3, 2019, without restrictions on publication date or language (see **S1** for complete strategy). [Methods]

A total of 3,404 titles and abstracts, and subsequently, 972 full-text articles were screened; 205 met our eligibility criteria and were included in the review (**Fig 1**). [Results]

**Reviewers' comments:**

**Reviewer #1: General comments:**

The study idea about the socioeconomic impact of orthopaedic trauma is interesting and valuable, furthermore to be studied in a systematic review and meta- analysis study design elaborates more information in a wider and fruitful way.

In general, it’s a well written manuscript according to the PRISMA guidelines. I have provided some remarks below.

**Response:** We thank the reviewer for their complimentary remarks.

Abstract:

- The abstract is informative but the number of the included studies is not mentioned, please add it.

**Response:** Thank you for identifying this oversight. We have added the number of included studies in the revised manuscript.

**Revision:** Two-hundred-five studies met the eligibility criteria. [Abstract]

Methods:

Under: Data Synthesis and Analysis section, in line 122: linguistic correction; “ fractures types” to be corrected to fractures’ types or types of fractures.

**Response:** We have corrected the sentence as suggested.

**Revision:** The types of fractures were defined using the Arbeitsgemeinschaft für Osteosynthesefragen (AO)/ Orthopaedic Trauma Association (OTA) Fracture and Dislocation Classification Compendium, 2018 [11].

Results:

All tables and figures are presented in a clear and informative way, with few comments:

- Table (1): in study types: please clarify in details the other types and insert it in the table or as a footnote under the table.

**Response:** We have added the study types included in the “other” category as a footnote to Table 1.

**Revision:** Other study types included four quasi-experimental studies, two longitudinal studies, and two cost-effectiveness studies.

- One of the study characteristics included is fracture location of the study, in some studies more than one location were found. This characteristic is better to be included under patient characteristics as patient wise, to be included in table (2) after mechanism of injury.

**Response:** We agree that the fracture location would be more informative as patient-level data. However, many of the studies had broad eligibility criteria that included several fracture locations but did not report the number of patients that had fractures in each specific location in their manuscript. We were, therefore, unable to devise precise estimates on the number of individual patients that sustained specific fractures in our meta-analysis dataset.

- In table (3) about Socioeconomic Outcome Measures, under data collection methods: clarify in details the details in the table or as a footnote.

**Response:** The “other” type of data collection method should have been more accurately coded as “not specified”. We have updated the table accordingly.

- The term “days absent from work” could be replaced by absenteeism days from work all over the manuscript.

**Response:** We thank the reviewer for their suggestion and have updated “days absent from work” to “absenteeism days from work” throughout the manuscript.

Discussion:

- Is well written and covering all items.

- In line 309: What’s meant by prohibited? Please, replace with suitable term.

**Response:** We have changed “prohibited” to “not possible” in the discussion section.

**Revision:** Other subgroup analyses were not possible due to inconsistent reporting of potential confounders, such as the severity of the injury, patient comorbidities, the type of pre-injury employment, and legal adjudication for compensation.[Discussion]

**Reviewer #2:** The Manuscript The Socioeconomic Impact of Orthopaedic Trauma: A Systematic Review and Meta-Analysis addresses an extremely important issue of orthopedic health care: although due to aging societies, age related diseases are more and more frequent, orthopedic Trauma is jeopardizing patients in their best years. By that the quantitative description of work loss etc. is for outstanding interest for the scientific Society and I strongly recommend to publish this study.

**Response:** We thank the reviewer for their gracious feedback.

My only Question would be an Annotation in the discussion: the follow up periods are significantly different between prospective and retrospective studies, which somehow reflects a currently taking place paradigma Change in the operative disciplines a Little bit away from the prospective studies towards the large retrospective Register studies. Hence, it would be good to add one or two sentetnce in the discussion, that the Long term follow ups in orthopedic surgery require probably more big Register studies in the future. Beside this, tha manuscript has my full support for publication and I want to congratulate the authors to their work

**Response:** We agree with the reviewer that registries present an incredible opportunity to obtain long-term, population-level estimates of the socioeconomic effects of fractures. We have added that important point to the discussion, but also included the caveat that to estimate socioeconomic effects, health registry data must reliably collect socioeconomic measures or have identifiers that can be linked to socioeconomic registries.

**Revision:** The increased availability of large registry data presents an opportunity for long-term, population-level estimates of the socioeconomic effects of fractures. However, to realize this opportunity, socioeconomic data must be routinely and reliably collected in health data registries, or health data registries must include identifiers than can be linked to socioeconomic registries.
